# Proteomic biomarkers in seminal plasma as predictors of reproductive potential in azoospermic men

**DOI:** 10.3389/fendo.2024.1327800

**Published:** 2024-04-09

**Authors:** Daniela Fietz, Raouda Sgaier, Liza O’Donnell, Peter G. Stanton, Laura F. Dagley, Andrew I. Webb, Hans-Christian Schuppe, Thorsten Diemer, Adrian Pilatz

**Affiliations:** ^1^ Department of Veterinary Anatomy, Histology and Embryology, Justus Liebig University Giessen, Giessen, Germany; ^2^ Centre for Reproductive Health, Hudson Institute of Medical Research, Clayton, VIC, Australia; ^3^ Department of Urology, Pediatric Urology and Andrology, Justus Liebig University Giessen, Giessen, Germany; ^4^ Department of Molecular and Translational Sciences, Monash University, Clayton, VIC, Australia; ^5^ Advanced Technology and Biology Division, Walter and Eliza Hall Institute, Parkville, VIC, Australia; ^6^ Department of Molecular and Translational Sciences, School of Clinical Sciences, Monash University, Clayton, VIC, Australia

**Keywords:** azoospermia, proteomics, diagnosis, testicular failure, seminal plasma, biomarker

## Abstract

**Introduction:**

Azoospermia, characterized by an absence of sperm in the ejaculate, represents the most severe form of male infertility. While surgical sperm retrieval in obstructive azoospermia (OA) is successful in the majority of cases, patients with non-obstructive azoospermia (NOA) show retrieval rates of only about 50% and thus frequently have unnecessary surgery. Surgical intervention could be avoided if patients without preserved spermatogenesis are identified preoperatively. This prospective study aimed to discover biomarkers in seminal plasma that could be employed for a non-invasive differential diagnosis of OA/NOA in order to rationalize surgery recommendations and improve success rates.

**Methods:**

All patients signed written informed consent, underwent comprehensive andrological evaluation, received human genetics to exclude relevant pathologies, and patients with azoospermia underwent surgical sperm retrieval. Using label-free LC-MS/MS, we compared the proteomes of seminal plasma samples from fertile men (healthy controls (HC), n=8) and infertile men diagnosed with 1) OA (n=7), 2) NOA with successful sperm retrieval (mixed testicular atrophy (MTA), n=8), and 3) NOA without sperm retrieval (Sertoli cell-only phenotype (SCO), n=7). Relative abundance changes of two candidate markers of sperm retrieval, HSPA2 and LDHC, were confirmed by Western Blot.

**Results:**

We found the protein expression levels of 42 proteins to be significantly down-regulated (p ≤ 0.05) in seminal plasma from SCO NOA patients relative to HC whereas only one protein was down-regulated in seminal plasma from MTA patients. Analysis of tissue and cell expression suggested that the testis-specific proteins LDHC, PGK2, DPEP3, and germ-cell enriched heat-shock proteins HSPA2 and HSPA4L are promising biomarkers of spermatogenic function. Western blotting revealed a significantly lower abundance of LDHC and HSPA2 in the seminal plasma of men with NOA (SCO and MTA) compared to controls.

**Discussion:**

The results indicate that certain testis-specific proteins when measured in seminal plasma, could serve as indicators of the presence of sperm in the testis and predict the success of sperm retrieval. Used in conjunction with conventional clinical assessments, these proteomic biomarkers may assist in the non-invasive diagnosis of idiopathic male infertility.

## 1 Introduction

Infertility affects approximately 15% of couples of reproductive age in Western societies with almost equivalent male and female contributions (1, 2). 4-7% of couples seek infertility medical treatment, but it is fair to assume that infertility, especially in males, is underreported (2, 3). Azoospermia, or the complete absence of sperm from the ejaculate, is the most severe form of male infertility that affects nearly 2% of men in the general population and is responsible for 5-20% of male infertility cases (4, 5). Azoospermia can be clinically categorized as obstructive azoospermia (OA) and non-obstructive azoospermia (NOA). While OA is caused by a physical obstruction in the male reproductive tract at the level of the rete testis, epididymal duct, vas deferens, seminal vesicles, or prostate (1), NOA accounts for about 90% of azoospermia cases and results from varying degrees of testicular failure (4, 6). In NOA, histological evaluation of testis biopsies may reveal different phenotypes with variable degrees of disorders in spermatogenesis. Hypospermatogenesis is also referred to as mixed testicular atrophy (MTA), and is characterized as qualitatively intact spermatogenesis in some areas of the biopsy, usually combined with various degrees of spermatogenic impairment that differ between seminiferous tubules. Sertoli cell only phenotype (SCO) is characterized by a complete depletion of germ cells in all seminiferous tubules examined (7, 8). Accurate diagnosis of azoospermia is of prime importance to rationalize the treatment of azoospermic men (9). This is because patients with OA have a good surgical sperm retrieval rate (>90%), and in selected cases, reconstructive surgery to facilitate natural conception is possible (10). On the other hand, the chance for positive sperm retrieval in men with NOA is only about 50% (11). Due to the limited predictive value of classical parameters for positive sperm retrieval such as age, hormones, and testicular volume (12, 13), testicular biopsy is still the gold standard method to differentiate between OA and NOA and enables testicular sperm retrieval (9, 14, 15). It would be desirable to identify patients who do not have ongoing spermatogenesis preoperatively to avoid a low-success-rate surgical intervention. Several studies reported on the discovery of new biomarkers for the differential diagnosis of the origin of azoospermia in semen, but often miss adequate testicular histology (16–19). Seminal plasma contains thousands of tissue-specific proteins at relatively high concentrations, making it exceptionally reflective of the entirety of the male reproductive organs and a promising source of markers reflecting sub-/infertility (20, 21). The aim of this study was to distinguish the proteomic profiles of seminal plasma from men diagnosed with different forms of azoospermia compared to healthy controls and to identify biomarkers for male reproductive function using a label-free quantitative mass spectrometry-based proteomics approach.

## 2 Materials and methods

### 2.1 Patients and ethics

In this prospective study from 2017 to 2020, azoospermic patients underwent an extensive clinical examination including hormonal profiling, genetic analysis (karyotype, AZF deletions, CFTR mutations) and detailed medical history as reported by (22). The etiology of azoospermia was determined by histological scoring of testicular tissue collected during surgical testicular sperm extraction (TESE/m-TESE) as described by Fietz and Kliesch (8). Twenty-two patients with different forms of azoospermia (OA n=7, MTA n=8, SCO n=7) and eight fertile controls (healthy controls, HC) without genetic pathologies (AZF deletions, CFTR mutations, abnormal karyotype) were selected for this comprehensive analysis. Patient demographics and clinical parameters are provided in **Supplementary Tables 1**, **2**. All participants provided written informed consent for the collection and use of semen samples for research purposes. This study was approved by the local institutional review board (Ref. No. 26/11).

### 2.2 Sample preparation for mass spectrometry analysis

Semen samples were collected by masturbation after an abstinence period of 2 to 7 days according to WHO (23) recommendations and left to liquefy at room temperature (RT) for 1-2 h. After liquefaction, semen samples were centrifuged for 10 min at 13000 g. The supernatants (seminal plasma) were subsequently aliquoted in 2 mL polypropylene vials and kept at -80°C pending analysis. Total protein content was estimated using the Pierce BCA Protein Assay (Cat. # PI23227). Sample preparation was conducted as described in Dagley et al. (24). Seminal plasma samples were thawed on ice and diluted in MilliQ water. From each sample, a volume equivalent to 30 µg of total protein content was pipetted into a numbered tube. 12 µl of SDS lysis buffer (5% SDS/50 mM Tris-HCl, pH 7.5/50 mM Tris(2-carboxyethyl) phosphine-hydrochloride (pH 7), 200 mM 2-chloroacetamide) was added to each diluted sample. Samples were then boiled for 3 min at 95°C and left to cool at RT for 5 min. Carboxy agarose beads (PureCube Carboxy MagBeads, Cube Biotech) were pre-washed twice in 500 µl of MilliQ water and then reconstituted in an equal volume of water. 20 µl of the Carboxy agarose beads solution was pipetted into each sample tube, and neat acetonitrile was added resulting in a final 70% v/v. The sample/beads mixture was then incubated at RT in a ThermoMixer C (Eppendorf) at 400 rpm for 20 min. Next, the samples were placed on a magnetic rack for 2 min causing the protein-covered beads to adhere to the bottom of the wells/tubes. The beads were washed twice with 200 µl of 70% ethanol and once with 200 µl of neat acetonitrile. After lyophilization, 50 µl of a digestion buffer containing trypsin and lys-C was added to each sample and the mixture was sonicated in an ultrasonic bath for 2 min before incubation in ThermoMixer C for 1 hour at 37°C, shaking at 400 rpm. The samples were again placed on the magnetic rack and 50 µl of MilliQ water was added to each well and the supernatants were collected after 1 min of sonication. Offline HPLC fractionation was performed as described in O’Donnell et al. (25). C18 stage tips were prepared by adding two plugs of 3M Empore resin (#2215) to 200ul plastic pipette tips. After equilibration using a series of polar solvents, the individual peptide samples were loaded into the tips and centrifuged at 800 x g for 2 min until only a small volume remained on top of the resin plug. After two washes with 5% formic acid, the tips were placed into Lobind Eppendorf tubes, and the peptides were eluted with 50 µl of 50% acetonitrile/5% formic acid followed by 50 µl of 80% acetonitrile/5% formic acid for 1-2 min at 800 x g. The eluate was transferred into mass spectrometry vials, frozen at 80°C then lyophilized down to dryness in preparation for MS analysis.

### 2.3 LC-MS/MS analysis

Eluted peptide mixtures were analyzed by reverse-phase liquid chromatography-tandem mass spectrometry. Samples were separated through a C18-fused silica column (O.D. 75μm) packed into an emitter tip (IonOpticks, Fitzroy, VIC, Australia) by reverse-phase chromatography using a nano-flow HPLC (M-class; Waters, Wilmslow, UK). The peptide mixtures were loaded directly onto the column at a constant flow rate of 400 nL/min with 0.1% formic acid in Milli-Q water and eluted with a 99.9% acetonitrile and 0.1% formic acid linear gradient (from 2 to 34%, 90 min). The chromatography setup was connected to an Impact II UHR-QqTOF mass spectrometer (Bruker, Billerica, MA, USA) via a CaptiveSpray source and nanoBooster at 0.20 Bar using acetonitrile. Data-dependent acquisition of mass spectra occurred by switching between MS and MS/MS scans automatically using a 4 Hz rate for MS1 spectra scans and 8–20 Hz for MS/MS scans according to precursor intensity. The set mass range for spectra acquisition was 200–2000m/z. Peptide fragmentation was achieved by collision-induced dissociation.

### 2.4 Data processing and analysis

Raw MS f were processed with MaxQuant (version 1.5.8.3). MS/MS spectra were matched using the Andromeda search engine to peptide sequences from the UniprotKB/Swiss-Prot Homo sapiens reference proteome (July 2019). The false discovery rate (FDR) was controlled by an automatically generated reverse decoy database, consistent with trypsin digestion and allowing a maximum of two missed cleavages. Differential expression analysis was performed using the LFQ-Analyst platform (https://bioinformatics.erc.monash.edu/apps/LFQ-Analyst/) (26). To adjust for multiple testing, p-values were corrected using the Benjamini-Hochberg procedure. Proteins were deemed to have significant differential expression if the p-values ≤ 0.05. Mass spectrometry proteomics data were deposited in the ProteomeXchange Consortium via the PRIDE (27) partner repository with the dataset identifier PXD045519.

### 2.5 Gene ontology and pathway analysis

Gene names of the identified proteins were matched to a list of 2237 testis-enriched and 94 epididymis-enriched proteins downloaded from the Human Protein Atlas database (https://www.proteinatlas.org/). The molecular functions of the significantly differentially expressed proteins were determined using the Gene Ontology (GO) enrichment tool (http://geneontology.org/) powered by the PANTHER (Protein ANalysis THrough Evolutionary Relationships) Classification System and KEGG BRITE, Kyoto Encyclopedia of Genes and Genomes’ reference database for functional hierarchies of genes and proteins. Associated molecular pathways were explored via the KEGG PATHWAY tool. Cytoscape (version 3.6.0) was used to retrieve the edges/interactions between differentially expressed proteins from the STRING database (www.string-db.com) and visualize the network.

### 2.6 Western blot

Seminal plasma samples from fertile men (healthy controls, HC, n=8), mixed testicular atrophy (MTA, n=8), and Sertoli cell only (SCO, n=8) patients were used for WB analysis. The relative abundances of LDHC and HSPA2 in the seminal plasma of healthy fertile men, MTA, and SCO samples were measured by immunoblotting. Seminal plasma samples were thawed on ice and vortexed lightly before dilution using Bolt LDS Sample Buffer (1X) (Life Technologies, #B0007) and heated at 70°C for 10 min after the addition of a reducing agent (Life Technologies, #B0009). The samples were subsequently separated on Bolt 4-12% Bis-Tris Plus Gels (Invitrogen) under reducing conditions and subsequently transferred onto PVDF membranes (Immobilon-P 0.45 µm, Merck, IPVH00010). The membranes were incubated for 1h at RT in Blocking Buffer (1% BSA/TBS, 0.05% Tween), after which they were probed with the recommended dilutions of anti-LDHC and anti-HSPA2 polyclonal antibodies produced in Rabbit (Invitrogen, #PA5-30079 and #PA5-29083). After 3 washes of 10 min each with washing buffer (TBS/0.05% Tween), the membranes were incubated in 1:10,000 Goat anti-Rabbit IgG (H+L) Cross-Adsorbed Secondary Antibody-HRP conjugate (Invitrogen, Cat. # G-21234) in 5% skim milk powder, PBS 0.1% Tween for 1h at RT. Each membrane was washed 4 times before adding 2 mL Lumi-LightPLUS Western Blotting Substrate (Merck, #12015196001). Bands were visualized in a ChemiDoc XRS+ imaging system. Image Lab 6.0 (BIO-RAD) was used to acquire blot images and quantify protein bands. Statistical significance was determined using the Mann-Whitney test (GraphPad Prism 8). The samples used in this experiment are not the same as those analyzed by mass spectrometry, although they come from the same patient cohort and have similarly verified diagnoses based on histological evaluation of testicular biopsies.

### 2.7 Statistical analysis

The demographics and characteristics of the patients with different forms of azoospermia are provided as medians and interquartile range (IQR). Analyses were performed using SPSS 27 for Windows (IBM GmbH, Ehningen, Germany).

## 3 Results

### 3.1 Identification and relative quantification of seminal plasma proteins by mass spectrometry

**Figure 1:**
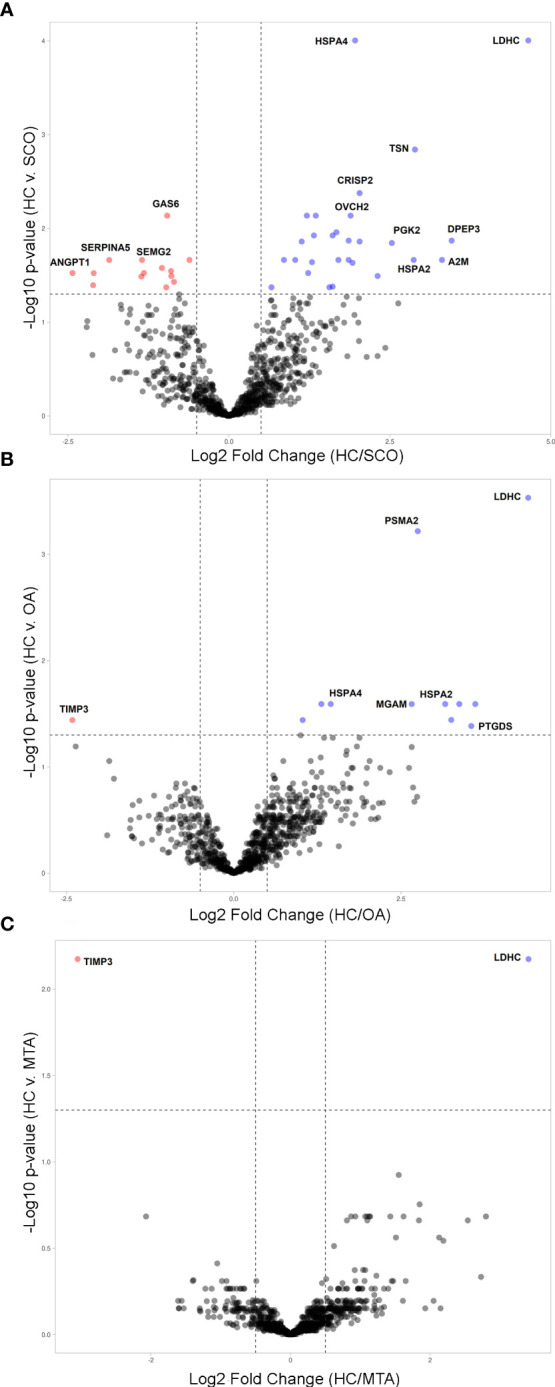
Volcano plots illustrating the differentially expressed proteins (DEPs) by type of azoospermia as identified by quantitative MS analysis. **(A)** in SCO, **(B)** in OA and **(C)** in MTA compared with HC. Y-axis represents negative log10 of adjusted p-values. Proteins were deemed differentially regulated if the log2 fold change in protein expression was greater than 1.5-fold (0.58, blue) or less than 0.66 (red) and a –log10 p-value (with Benjamini-Hochberg correction) ≥ 1.3, equivalent to a p-value ≤ 0.05. Proteins with a positive Blue dots represent DEPs of higher abundance in HC seminal plasma compared to the azoospermia groups (SCO, OA, MTA), and red dots represent significant proteins that are less abundant in the HC group compared to azoospermia samples. The plots were generated VolcaNoseR (https://huygens.science.uva.nl/VolcaNoseR/).

To determine changes in seminal plasma protein expression in fertile men vs infertile men with specific clinical phenotypes, seminal plasma samples from healthy controls (HC, n=8) and men diagnosed with OA (n=7), MTA (n=8), and SCO (n=8), respectively, were analyzed by label-free LC-MS/MS. A total of 917 proteins were quantified across all samples. Given the stringent statistical analysis used to estimate protein ratios, proteins with p-values ≤ 0.05 and fold changes (FCs) ≥ 1.5 reflecting an absolute scale were considered significant (**Supplementary Table 3**). Notable changes in protein expression were seen when comparing the healthy controls (HC) with the SCO and OA groups. Proteins were deemed differentially regulated if the log2 fold change in protein expression was greater than 1.5-fold (0.58, blue dots in **Figure 1**) or less than 0.66 (red dots in **Figure 1**) and a –log10 p-value (with Benjamini-Hochberg correction) ≥ 1.3, equivalent to a p-value ≤ 0.05. The HC/SCO comparison showed a significant change in the abundance of a total of 42 proteins (**Figure 1A**). Fold changes and p-values corresponding to these 42 proteins are shown in **Table 1**. Of these, 28 proteins exhibited down-regulated expression in SCO compared to HC and 14 proteins were found to have up-regulated expression (**Table 1**). Twelve proteins were discovered to have significantly different protein expression in the OA group relative to HC (**Figure 1B**), with the majority (11/12) having more than 2-fold higher expression in the HC group compared to OA (**Table 2**). These include PTGDS (FC HC/OA= 11.71, p=0.041) and MGAM (FC HC/OA= 6.32, p=0.025). In the HC/MTA comparison, LDHC and TIMP3 were the only proteins to exhibit a significant difference in protein abundance (FC HC/MTA= 10.63 and 0.12, respectively) (**Figure 1C**). No significant differences in protein expression were revealed in the statistical comparisons between the three azoospermia groups (**Supplementary Table 3**), and several proteins showed similar fold changes in HC/OA and the HC/SCO comparisons, namely LDHC, A2M, DPEP3, HSPA2, HSPA4, and HSP90AA1 (**Tables 1**, **2**). Overall, the comparison between HC/SCO revealed the highest number of differentially expressed proteins, with the fewest seen in the HC/MTA comparison.

**Table 1:** Proteins with significantly different abundances in Sertoli cell only (SCO) patients’ seminal plasma relative to healthy fertile controls (HC), arranged by decreasing order of fold change (HC/SCO); p-values were corrected using the Benjamin-Hochberg procedure.

Protein IDs	Gene Name	Protein Names	Fold change HC/SCO	Adjusted p-value HC vs SCO
P07864	LDHC	L-lactate dehydrogenase C chain	25.11	<0.0001
Q9H4B8	DPEP3	Dipeptidase 3	11.00	0.0135
P01023	A2M	Alpha-2-macroglobulin	9.92	0.0217
E9PGT1	TSN	Translin	7.41	0.0014
P54652	HSPA2	Heat shock-related 70 kDa protein 2	7.31	0.0217
P07205	PGK2	Phosphoglycerate kinase 2	5.78	0.0143
P04080	CSTB	Cystatin-B	4.96	0.0322
P16562	CRISP2	Cysteine-rich secretory protein 2	4.08	0.0042
Q9Y266	NUDC	Nuclear migration protein nudC	4.08	0.0138
P34932	HSPA4	Heat shock 70 kDa protein 4	3.89	<0.0001
P15374	UCHL3	Ubiquitin carboxyl-terminal hydrolase	3.78	0.0233
A0A087X1V8	OVCH2	Ovochymase-2	3.71	0.0073
P50990-2	CCT8	T-complex protein 1 subunit theta	3.63	0.0135
Q9NZJ9	NUDT4	Diphosphoinositol polyphosphate phosphohydrolase 4	3.63	0.0217
O95757	HSPA4L	Heat shock 70 kDa protein 4L	3.25	0.0217
B3GLJ2	PATE3	Prostate and testis expressed protein 3	3.18	0.011
Q99832	CCT7	T-complex protein 1 subunit eta	3.05	0.0418
Q9Y265	RUVBL1	RuvB-like 1	3.05	0.0119
Q7L266	ASRGL1	Isoaspartyl peptidase/L-asparaginase	2.95	0.0425
Q02952-3	AKAP12	A-kinase anchor protein 12	2.55	0.0073
Q9UQ80	PA2G4	Proliferation-associated protein 2G4	2.50	0.0119
F8VY35	NAP1L1	Nucleosome assembly protein 1-like 1	2.45	0.0229
P37802	TAGLN2	Transgelin-2	2.35	0.0299
P07900	HSP90AA1	Heat shock protein HSP 90-alpha	2.31	0.0073
J3KQ45	TGOLN2	Trans-Golgi network integral membrane protein 2	2.19	0.0138
P80723	BASP1	Brain acid soluble protein 1	2.04	0.0217
P08238	HSP90AB1	Heat shock protein HSP 90-beta	1.81	0.0217
H0Y7A7	CALM2	Calmodulin	1.58	0.0425
P42785	PRCP	Lysosomal Pro-X carboxypeptidase	0.65	0.0217
G5E9W0	PLA1A	Phospholipase A1 member A	0.55	0.0372
Q9GZN4	PRSS22	Brain-specific serine protease 4	0.54	0.0322
A0A0G2JNJ8	MUC6	Mucin-6	0.54	0.0285
Q14393-2	GAS6	Growth arrest-specific protein 6	0.51	0.0073
P02766	TTR	Transthyretin	0.51	0.0425
P05154	SERPINA5	Plasma serine protease inhibitor	0.49	0.0264
O60243	HS6ST1	Heparan sulfate 6-O-sulfotransferase 1	0.40	0.0299
Q02383	SEMG2	Semenogelin-2	0.39	0.0217
P35270	SPR	Sepiapterin reductase	0.39	0.0326
Q9P232	CNTN3	Contactin-3	0.28	0.0217
Q15904	ATP6AP1	V-type proton ATPase subunit S1	0.23	0.03
P35625	TIMP3	Metalloproteinase inhibitor 3	0.23	0.0403
Q15389-2	ANGPT1	Angiopoietin-1	0.19	0.0299

**Table 2:** Proteins with significantly different abundances in obstructive azoospermia (OA) patients’ seminal plasma relative to healthy fertile controls (HC), arranged by decreasing order of fold change (HC/OA); p-values were corrected using the Benjamin-Hochberg procedure.

Protein IDs	Gene Name	Protein Names	Fold change HC/OA	Adjusted p-value HC vs OA
P07864	LDHC	L-lactate dehydrogenase C chain	21.11	0.0003
P01023	A2M	Alpha-2-macroglobulin	12.21	0.0256
P41222	PTGDS	Prostaglandin-H2 D-isomerase	11.71	0.0412
J3KTF8	ARHGDIA	Rho GDP-dissociation inhibitor 1	10.34	0.0256
Q9H4B8	DPEP3	Dipeptidase 3	9.51	0.0361
P54652	HSPA2	Heat shock-related 70 kDa protein 2	8.94	0.0256
P25787	PSMA2	Proteasome subunit alpha type-2	6.73	0.0006
E7ER45	MGAM	Maltase-glucoamylase	6.32	0.0256
P78371	CCT2	T-complex protein 1 subunit beta	2.73	0.0256
P34932	HSPA4	Heat shock 70 kDa protein 4	2.48	0.0256
P07900	HSP90AA1	Heat shock protein HSP 90-alpha	2.04	0.0362
P35625	TIMP3	Metalloproteinase inhibitor 3	0.19	0.0362

### 3.2 Tissue specificity of differentially expressed proteins

**Figure 2:**
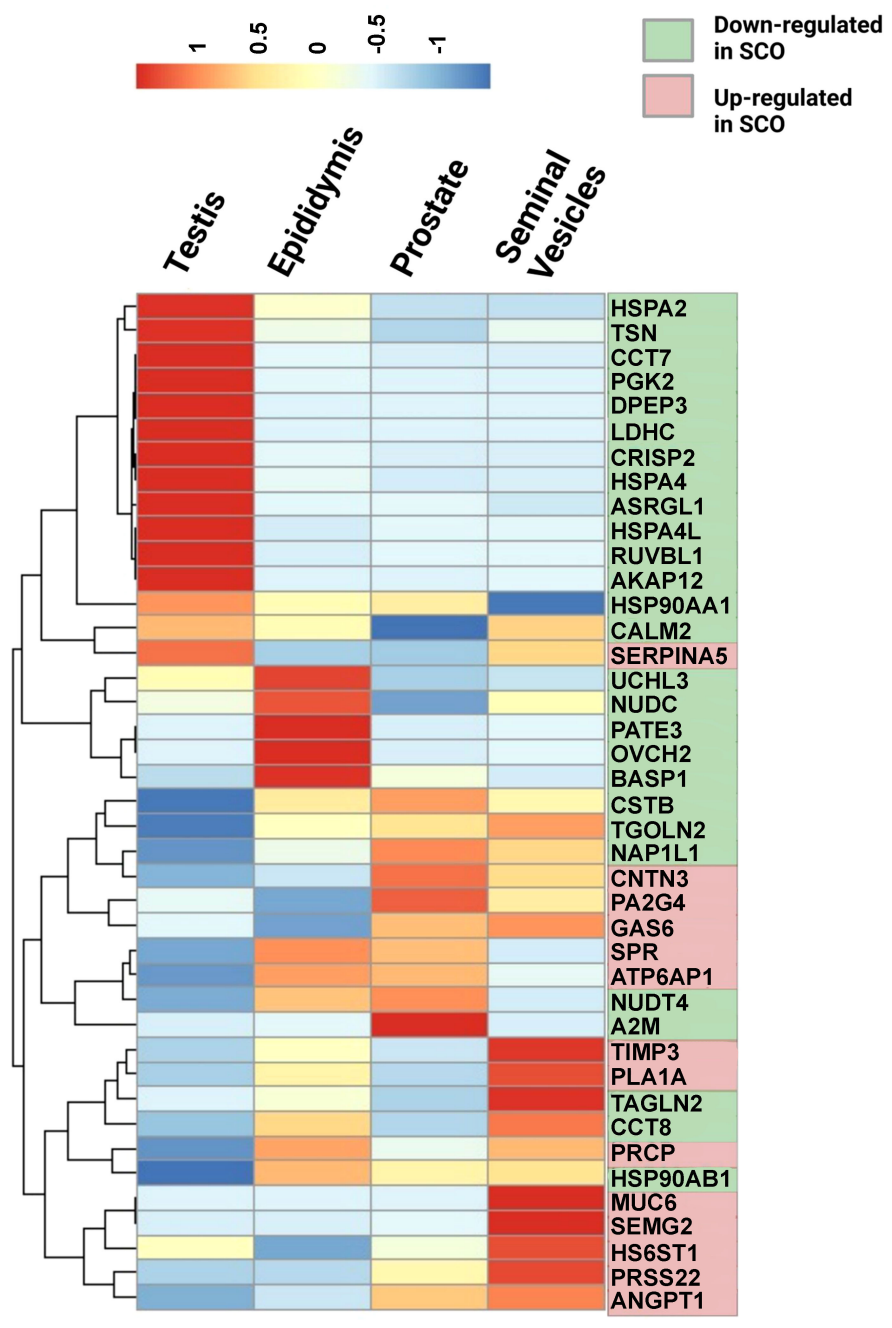
Relative mRNA expression in male reproductive tissues of 42 proteins that were significantly different in SCO seminal plasma compared to controls. mRNA expression data in the testis, epididymis, prostate, and seminal vesicles was extracted from the consensus dataset the Tissue Atlas database (www.proteinatlas.org). The heatmap was generated from scaled TPM (Transcript per Million) values using the “pheatmap” package in RStudio 1.4.1717-3. Hierarchical clustering was used to group proteins according to their expression pattern in male reproductive tissues (rows clustering distance measure = Euclidian). Scale: read counts for each gene were normalized across the four tissues to have mean value of 0 and standard deviation of 1.

The Human Protein Atlas (HPA) tissue database was queried to determine the cellular origin of differentially expressed proteins (DEPs). Tissue and single-cell specificity of DEPs in male reproductive organs are specified in the fourth column of **Supplementary Table 3**. To better understand SCO-induced gene expression changes in male tissues, we extracted mRNA expression levels of the 42 DEPs identified in the HC/SCO comparison from the HPA consensus dataset. Hierarchical clustering of this data (**Figure 2**) shows that proteins of higher abundance in SCO compared to the HC group are expressed more in the prostate and seminal vesicles (SEMG2, ANGPT1). By contrast, proteins of lower abundance in SCO are either testis-enriched/enhanced (e.g. PGK2, DPEP3, ASRGL1, HSPA4L), enriched in one or more germ cell types (e.g. HSPA2, CCT7) or epididymis-enriched (PATE3, OVCH2, and BASP1). Proteins that are significantly less abundant in OA compared to HC include epididymis-enhanced MGAM and testis-enhanced PTGDS (**Supplementary Table 3**). Differences in the abundances of these tissue-specific proteins in SCO are likely associated with organ dysfunction whereas in OA, proteomic differences are likely due to obstructed access to epididymal and testicular secretions.

### 3.3 Gene ontology and molecular functions of DEPs

**Figure 3:**
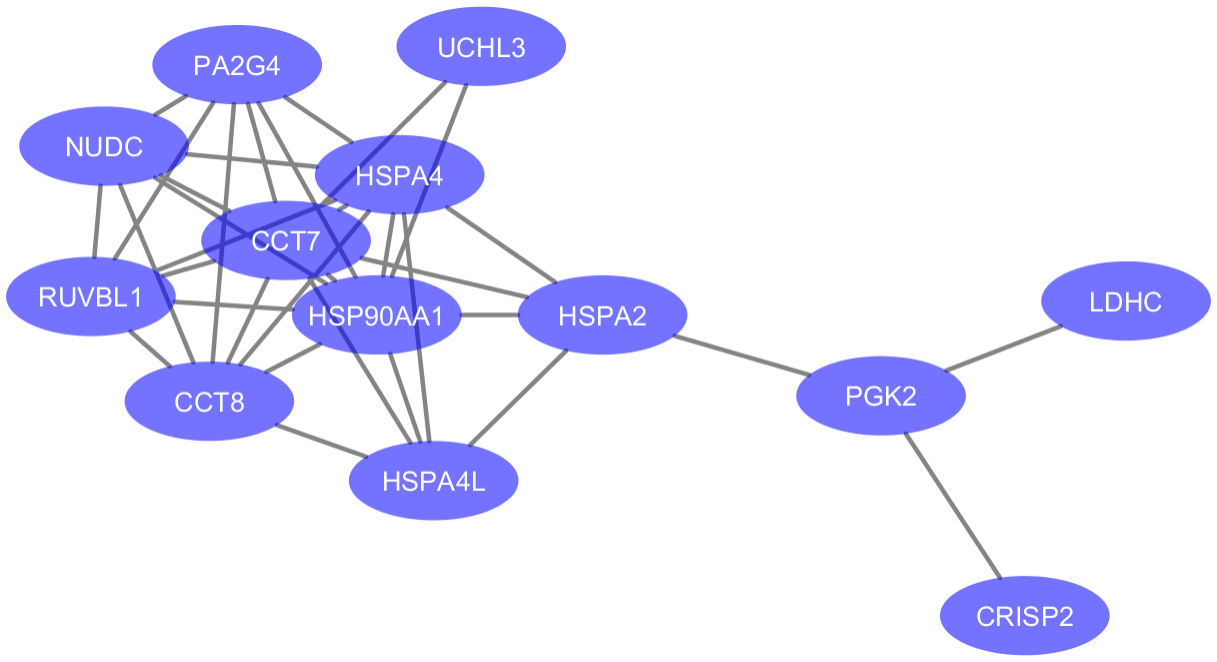
Protein-protein associations of 13 proteins downregulated in testicular failure (PPI enrichment p-value: 3.51e-09). The network was visualized in Cytoscape (version 3.6.0) using the STRING database (https://string-db.org/) to determine protein-protein interactions. Enriched gene ontology terms include “Protein Folding” (GO:0006457, FDR value = 0.0045) and “Unfolded protein binding” (GO:0051082, FDR value = 0.0011). Six proteins are part of the Chaperone complex and chaperone cofactor-dependent proteins STRING cluster (HSPA2, HSPA4, HSPA4L, CCT7, CCT8 and NUDC).

Since the depletion of testicular proteins in seminal plasma samples of OA is mainly due to the obstruction at the epididymal level, we focused on proteins with significant differential abundance in NOA relative to HC for the analysis of molecular functions and cellular pathways potentially linked to testicular failure. A functional analysis of the DEPs in MTA compared to HC was not feasible in light of their low number (n=2). The enriched GO terms were assessed for the 28 proteins significantly down-regulated in SCO compared to HC from **Table 1**. These proteins were enriched in biological processes and molecular functions associated with chaperone activity such as “protein folding” (GO:0006457), “chaperone-mediated protein complex assembly” (GO:0051131), “response to unfolded protein” (GO:0006986), and “unfolded protein binding” (GO:0051082). Besides heat-shock proteins HSPA2, HSPA4, and HSPA4L, proteins involved in these cellular processes include T-complex protein 1 subunits eta and theta (CCT7 and CCT8) all belonging to the CL:3015 STRING cluster Chaperone complex, and chaperone factor-dependent protein refolding. Network analysis using the STRING database (https://string-db.org/) generated a 12-node network with protein-protein interaction confidence scores >= 0.45 (**Figure 3**). Overall, proteins with lower abundance in SCO compared to HC fell into two main categories: products of germ cell-specific genes and proteins with a role in protein folding and complex formation.

### 3.4 Confirmation of MS findings using Western immunoblotting

**Figure 4:**
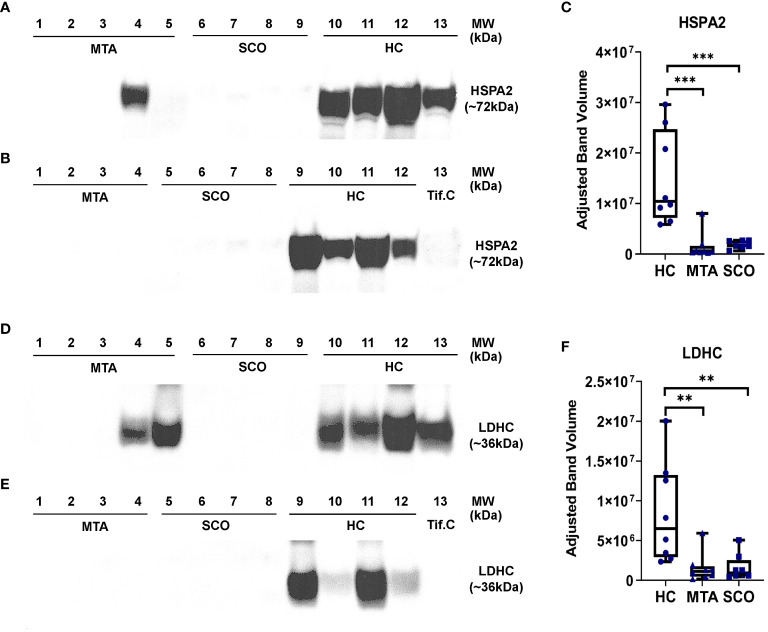
Relative quantitation of HSPA2 **(C)** and LDHC **(F)** in seminal plasma by Western immunoblotting. Seminal plasma samples from fertile men (healthy controls, HC, n=8), mixed testicuar atrophy (MTA, n=8), and Sertoli cell only (SCO, n=8) patients were resolved on 4-12% polyacrylamide gradient gels. Mw marker: SeeBlue Plus2 Pre-Stained Protein Standard (Life Technologies, #LC5925). A testicular interstitial fluid sample (Tif.C) was loaded as positive control. Bands corresponding to the Mw of HSPA2 **(A, B)** and LDHC **(D, E)** were visualized and their intensities were measured using Image Lab 6.0 (Bio-Rad) and corrected for total protein content. Statistical significance was determined by the Mann-Whitney test. ***p-value < 0.001; **p-value < 0.01. boxes represent median, 1st and 3rd quartiles, whiskers represent maximum and minimum.

LDHC and HSPA2 were selected as candidate biomarkers of spermatogenesis in NOA based on their relative abundance in seminal plasma measured by mass spectrometry and their high expression levels in germ cells. The relative abundances of LDHC and HSPA2 were measured in seminal plasma from healthy controls and from men with confirmed SCO or MTA diagnoses using Western Blot. Statistical significance was determined by the Mann-Whitney test. Densitometric analysis revealed a clear difference in LDHC and HSPA2 abundance between healthy controls and SCO and MTA groups (**Figure 4F**). LDHC was significantly decreased in SCO and MTA compared to the healthy control samples (p= 0.0025 and 0.0019, respectively), but no significant difference was observed between the SCO and MTA groups (**Figure 4F**). Similar results were observed with HSPA2, where there was a markedly higher abundance in HC compared to SCO and MTA (p= 0.0003 and 0.0006, respectively) but no difference was observed between SCO and MTA (**Figure 4C**). Several MTA and SCO samples appear to have below-detection concentrations of HSPA2 and LDHC (**Figures 4A, B, D, E**). These results confirm the MS findings that protein levels of germ cell-derived LDHC and HSPA2 in seminal plasma are reduced in NOA patients compared to fertile men, with a similar range of values in MTA and SCO.

## 4 Discussion

In this study, we used a label-free, quantitative mass spectrometry-based proteomics approach coupled with a stringent statistics pipeline to compare the protein repertoire of seminal plasma from patients diagnosed with OA or NOA, the latter stratified to mixed testicular atrophy (MTA) and Sertoli cell-only (SCO) to that of fertile men (healthy controls, HC) to investigate whether proteins in seminal plasma could be used to discriminate the presence of spermatogenesis in the testis. Respective testicular biopsies of seminal plasma samples analyzed were histologically phenotyped according to score count analysis according to (8) (**Supplementary Table 2**). While other studies have performed a proteomic comparison of seminal plasma between fertile and infertile men (28, 29), this study aimed to address some of the limitations of these earlier studies such as smaller sample sizes (n<=5/group), using samples from post-vasectomy men instead of patients with a defined OA phenotype, and NOA samples with unspecified testicular phenotype. Large-scale protein characterization methods have been applied to the discovery of diagnostic biomarkers (17, 19) and these techniques have evolved in recent times to produce techniques with higher resolution and throughput. Seminal plasma is a biological fluid that is routinely collected during work up of the infertile male, and thus analysis of the seminal plasma proteome holds promise for the discovery of markers of male infertility that could predict the outcome of sperm retrieval in azoospermic men (20, 28, 30). Despite some promising results from validation studies (31, 32) there are no defined clinical biomarkers in seminal plasma that can be used for the differential diagnosis of azoospermia prior to surgical intervention.

### 4.1 Proteins of lower abundance in the seminal plasma of men with obstructive azoospermia

Our quantitative proteomics analyses revealed a reduction of proteins of testicular origin (e.g., LDHC, DPEP3, and PGK2) in the seminal plasma of OA compared to fertile controls, which can be attributed to the physical obstruction of the ductus deferens preventing spermatozoa and testicular secretions from passing into the seminal fluid. Prostaglandin-H2 D-isomerase (PTGDS) is expressed in all male tissues, yet it was significantly decreased only in OA seminal plasma compared to fertile controls. This finding is supported by previous studies suggesting that PTGDS is promising biomarker of obstructive azoospermia (33–36). Maltase-glucoamylase (MGAM) was also reduced in OA seminal plasma compared to fertile controls, and was previously reported to be of significantly lower abundance in the seminal plasma of men after vasectomy (28), suggesting that it could be useful for assessing the presence of sperm. We noted that the abundance of PTGDS and MGAM in the seminal plasma of NOA (MTA and SCO) was not significantly different to healthy controls. The reduced ability of PTGDS to discriminate NOA could be due to the fact that it is widely expressed in most testicular cells and that it is highly expressed in the epididymis (21, 34). Taken together, our results are consisted with the hypothesis that the epididymis contributes most of the PTGDS and MGAM measured in seminal plasma and suggest that these two proteins could potentially be used as markers of obstruction in the male reproductive tract.

### 4.2 Up-regulated expression of accessory gland proteins in SCO

There were several proteins that exhibit significant increased protein expression in SCO seminal plasma relative to the control including seminal-vesicle enriched Angiopoietin-1 (ANGPT1), Phospholipase A1 member A (PLA1A), and Semenogelin II (SEMG2) (Tissue Atlas, https://www.proteinatlas.org/). Semenogelins I and II (SEMG1, SEMG2) are involved in semen coagulation and liquefaction and are the predominant proteins in seminal plasma; they are secreted principally by the seminal vesicles, with low expression in the rest of male tissues. Increased seminal plasma levels of SEMG1 and SEMG2 have been reported in men with infertility (37). SERPINA5 was also increased in SCO seminal plasma compared to healthy controls. This protein degrades SEMG1 and SEMG2 during the transfer of spermatozoa to the female reproductive tract, and its inactivation in seminal plasma could be linked to infertility (38, 39). The reason why the SCO phenotype is associated with a significant up-regulation in the expression of seminal vesicle-expressed proteins is unclear, but could be due to a loss of testis-derived regulatory factors that impact on accessory gland protein secretion. Further research is needed to establish a link between SCO and the increased expression of SEMG2 and SERPINA5 and to investigate their roles as potential biomarkers of the SCO phenotype.

### 4.3 Chaperone and heat shock proteins exhibit decreased expression in SCO seminal plasma

Unlike in patients with OA, the testis does contribute proteins to the seminal plasma in NOA patients and thus the seminal plasma proteome can be reflective of testicular function. The marked reduction in the abundance of several chaperones in SCO seminal plasma is likely caused by their reduced expression in the seminiferous epithelium. The HSP 70 kDa protein family’s role in male germ cell development and fertility has been long established (40–42). HSPA2 is involved in post-testicular remodeling of the sperm head, necessary for oocyte recognition and binding and essential for the progression of spermatogenesis, both in meiosis and germ cell differentiation (43, 44). HSPA4 acts as a co-factor of other 70 kDa HSPs as part of the cytosolic protein folding mechanism (45). In a previous study of seminal plasma proteome by mass spectrometry analysis, HSPA4L was not detected in NOA, but was quantifiable in the seminal plasma of fertile men (46). Moreover, a decreased expression of HSPA4L was positively correlated with poor sperm parameters (47). Cui et al. also reported heat shock protein HSP 90-alpha (HSPA90AA1) among the proteins with reduced expression in seminal plasma from NOA patients (46). We detected the chaperonin proteins CCT7 and CCT8 to exhibit a significant reduction in protein expression in SCO seminal plasma, these have been reported to play a role in human male gametogenesis (48). Disease-specific pathways identified by querying DAVID and reactome.org/PathwayBrowser largely correspond to the previously indicated GO terms (protein folding, chaperone-mediated protein complex assembly, response to unfolded protein). Other enriched pathways such as “cellular response to heat stress”, “uptake and actions of bacterial toxins” and viral infection pathways were identified due to the overrepresentation of heat shock proteins, which are implicated in numerous signaling pathways, but have distinct functions in spermatogenic cells. In summary, our analysis shows that molecular chaperones and heat-shock proteins are likely contributed to the plasma proteome by germ cells and have reduced expression in seminal plasma from NOA patients revealing SCO. These proteins could be potential biomarkers of spermatogenic output.

### 4.4 Germ cell-specific proteins in seminal plasma as biomarkers of spermatogenic status in NOA

Quantitative proteomics data from our mass spectrometry-based analysis revealed that a number of germ cell-enriched proteins (LDHC, DPEP3, PGK2, HSPA2, and HSPA4L) were markedly depleted in SCO seminal plasma, suggesting these are potential biomarkers of testicular failure. Several of these proteins exhibited lower abundance in SCO compared to HC and have confirmed or potential roles in spermatogenesis and male fertility in general. LDHC is a testis-specific isoenzyme from the lactate dehydrogenase family and is enriched in pachytene spermatocytes, round spermatids, and flagella of spermatozoa (49). The role of LDHC in lactate metabolism and sperm motility has been established in mice (50). LDHC has been previously shown to be undetectable or markedly decreased in the seminal plasma from men with NOA or after vasectomy (28, 29), and has been proposed to be a marker of male fertility based on an aggregation of proteomic, transcriptomic, and genomic data (51). Thus, our data adds to a growing body of evidence that LDHC in seminal plasma is a promising biomarker of spermatogenic function. The levels of DPEP3 and PGK2 were also reduced in SCO seminal plasma, and were reported as undetected in seminal plasma from NOA men in past studies (26). DPEP3, expressed primarily in spermatogonia and spermatocytes, is known to play a role in spermatogenesis linked to its metalloproteinase action on the ADAM protein family, and its activity in the human testis is modulated by a previously validated marker of male fertility, TEX101 (52, 53). The expression of PGK2 is specific to developing sperm, and to the spermatogenic glycolytic pathway required for sperm motility (54, 55). CRISP2 is also involved in sperm motility and spermiogenesis (56, 57) and has previously been shown to be significantly decreased in the seminal plasma of men with asthenozoospermia, teratozoospermia, and asthenoteratozoospermia (58). LDHC and HSPA2 were selected for immunoblotting for several reasons: These two proteins were among the most significantly differentially expressed proteins in SCO compared to HC, down-regulated 25.11-fold and 7.3-fold, respectively (**Table 1**). According to the single cell-type consensus dataset (Human Protein Atlas, proteinatlas.org), LDHC and HSPA2 are highly transcribed in germ cells, particularly in spermatocytes and early spermatids. These transcription levels would likely result in higher protein abundance, leading to better detectability and quantification in HC and MTA samples. Western Blotting results confirmed the findings of mass spectrometry analysis. However, more sensitive quantification methods to detect differences between the seminal plasma proteomes of MTA and SCO, would be needed for further analysis. Our analysis in seminal plasma from men with SCO revealed proteins with confirmed localization and function in spermatogenesis and potential significance in development of NOA as they were severely downregulated or even undetectable in this pathology. Taking this information and our results into account, these proteins can be promising biomarkers that could discriminate the spermatogenic potential of the testis.

There is emerging evidence that the levels of germ cell-specific proteins in seminal plasma can correlate with male reproductive potential in NOA patients (20, 31). However, specific candidate proteins differ greatly from one study to another, which represents a major hurdle in linking particular forms of azoospermia to specific seminal plasma biomarkers. The discrepancies between studies could be attributed to the high inter-individual variability in protein expression (59), differences in sample handling and analytical methods (60), inter-cohort variability, and the lack of down-stream confirmation studies which could determine which biomarkers are more promising for validation (61). We analyzed seminal plasma samples as well as testicular biopsies from histologically clearly defined OA and NOA samples to add valuable information on these proteins. An example of differential protein localization of HSPA2 in SCO compared to HC can be taken from **Supplementary Figure 1**. The application of standardized proteomic methods and statistical pipelines to large cohorts across multiple laboratories could help to define a consensus around biomarkers of male fertility in seminal plasma.

### 4.5 Limitations

While the strengths of the study are the precise clinical characterization of the patients examined and individual analyses of each sample, the number of patients is limited due to the labor-intensive and costly proteomic analysis. Comparison of putative biomarkers LDHC and HSPA2 by Western blotting (chosen because of the lack of commercially reliable ELISA assays for seminal plasma) confirmed MS findings but did not reveal differences between MTA and SCO. However, various testicular proteins exhibited significantly lower expression in SCO compared to HC, but were not different in MTA compared to HC, pointing to likely differences between SCO and MTA seminal plasma. Further studies are required to compare larger cohorts of different NOA patients to identify discriminatory proteins between MTA and SCO. Differences in gene expression in the testis between MTA and SCO might not be detectable at the protein level in seminal plasma because of the high dynamic range of seminal plasma and the relatively low abundance of germ-cell specific proteins. These limitations could be overcome by using more sensitive proteomics methods (e.g. data independent acquisition, DIA) to measure a range of candidate proteins (particularly those that are germ-cell enriched and reduced in SCO vs HC) in a larger number of samples.

### 4.6 Conclusion

In this quantitative proteomics study of human seminal plasma, we identified several proteins that are promising diagnostic biomarkers of spermatogenesis that could discriminate the spermatogenic potential of the human testis. Our results suggest that the seminal plasma of OA men lacks proteins of epididymal origin such as MGAM. In the absence of obstruction, the reduced abundance of testis-specific proteins LDHC, PGK2, and DPEP3 and heat shock proteins HSPA2 and HSPA4L in seminal plasma is indicative of severely impaired spermatogenesis. Further studies could define a threshold of protein abundance that defines the presence of post-meiotic germ cell proteins in the seminal plasma of azoospermic men that can discriminate SCO from MTA and indicate the chance of successful sperm retrieval. The clinical utility of these biomarkers should be investigated by evaluating their predictive value in larger scale studies across multiple cohorts, and whether antibody-based or targeted mass spectrometry-based assays are most appropriate should be investigated. The quantification of a selection of biomarkers of epididymal and testicular function in seminal plasma, coupled with routine hormonal investigation, could help direct the differential diagnosis of azoospermia in patients where testicular biopsy is the only diagnostic option.

## Data availability statement

The names of the repository/repositories and accession number(s) can be found here: ProteomeXchange; accession number: PXD045519. Project Webpage: http://www.ebi.ac.uk/pride/archive/projects/PXD045519. FTP Download: https://ftp.pride.ebi.ac.uk/pride/data/archive/2024/03/PXD045519.

## Ethics statement

The studies involving humans were approved by Ethics Committee, Faculty of Medicine, Justus Liebig University Giessen (Ref. No. 26/11). The studies were conducted in accordance with the local legislation and institutional requirements. The participants provided their written informed consent to participate in this study.

## Author contributions

DF: Conceptualization, Funding acquisition, Resources, Writing – original draft, Writing – review & editing. RS: Conceptualization, Investigation, Methodology, Validation, Visualization, Writing – original draft, Writing – review & editing. LO: Conceptualization, Writing – review & editing. PGS: Conceptualization, Formal analysis, Investigation, Writing – review & editing. LFD: Formal analysis, Investigation, Software, Writing – review & editing. AIW: Formal analysis, Investigation, Software, Writing – review & editing. H-CS: Resources, Writing – review & editing. TD: Conceptualization, Funding acquisition, Resources, Supervision, Writing – review & editing. AP: Conceptualization, Funding acquisition, Resources, Writing – review & editing.

## Supplementary material

Supplementary Figure 1 Immunohistochemistry of HSPA2 in human testicular biopsies showing normal spermatogenesis **(A)** and SCO **(B)**. **(A)** In normal spermatogenesis, HSPA2 protein is localized in germ cells, specifically in spermatocytes (arrowhead) and spermatids (arrow). **(B)** In SCO, there is no specific intratubular (*) staining detected. Immunohistochemistry, DAB staining, hematoxylin counterstain; primary magnification x40.
