# Supplementary figures and images for: Proteomic biomarkers in seminal plasma as predictors of reproductive potential in azoospermic men
Source: Front Endocrinol (Lausanne). 2024 Apr 9;15:1327800. doi: 10.3389/fendo.2024.1327800 (PMC11035875; doi:10.3389/fendo.2024.1327800)

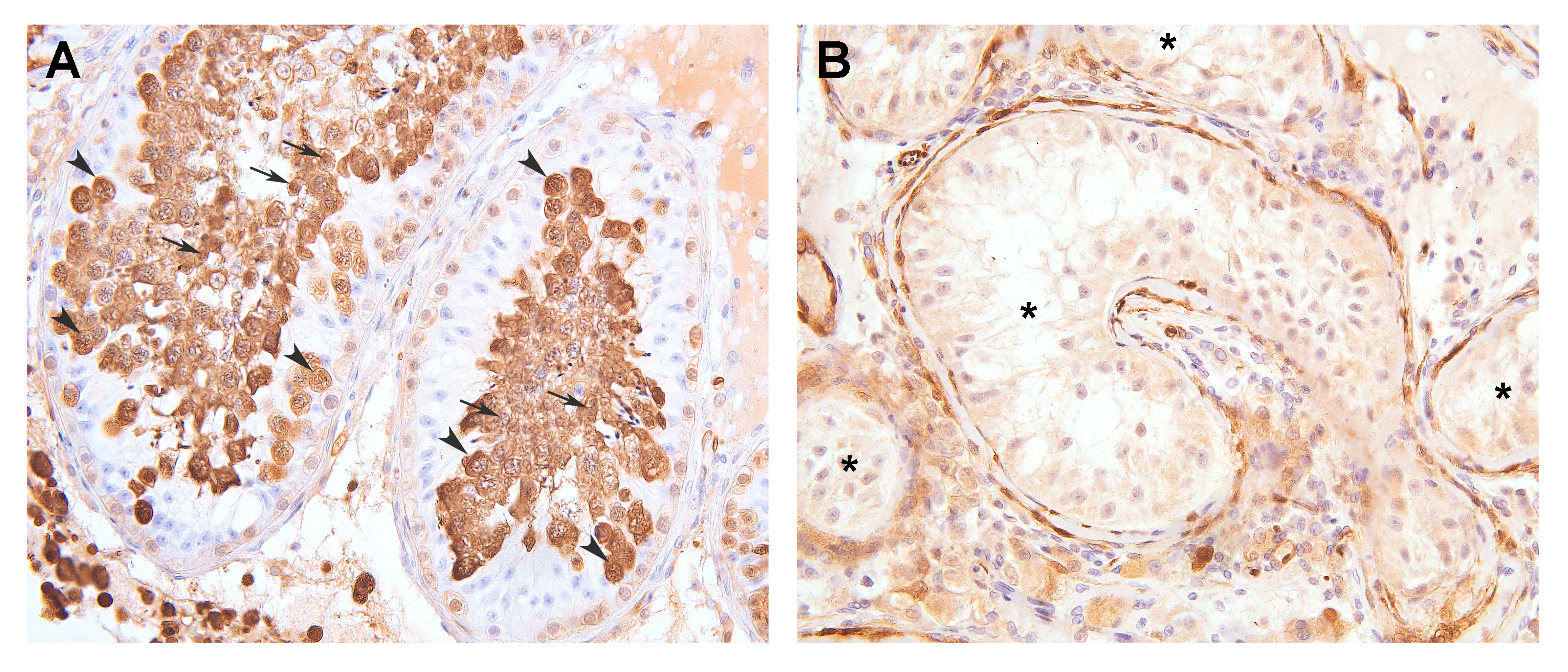

Supplement: Supplementary Figure 1 — Immunohistochemistry of HSPA2 in human testicular biopsies showing normal spermatogenesis (A) and SCO (B). (A) In normal spermatogenesis, HSPA2 protein is localized in germ cells, specifically in spermatocytes (arrowhead) and spermatids (arrow). (B) In SCO, there is no specific intratubular (*) staining detected. Immunohistochemistry, DAB staining, hematoxylin counterstain; primary magnification x40. [file Image_1.tif]
