# Supplementary material for: Proteomic biomarkers in seminal plasma as predictors of reproductive potential in azoospermic men
Source: Front Endocrinol (Lausanne). 2024 Apr 9;15:1327800. doi: 10.3389/fendo.2024.1327800 (PMC11035875; doi:10.3389/fendo.2024.1327800)
Supplement: Supplementary file 2 [file Table_1.docx]

| **Suppl. Tab. 1:**  Clinical data of the azoospermic patients based on testicular histology |
| --- |

|  |  | | |
| --- | --- | --- | --- |
|  | **Sertoli Cell Only syndrome**  **SCO** | **Obstructive Azoospermia**  **OA** | **Mixed Atrophy**  **MA** |
|  | n=7 | n=8 | n=7 |
| **Demographics** |  | | |
| Patient's age (years) | 33 (27-37) | 35 (28-48) | 34.5 (31-48) |
| Body height (cm) | 180 (175-180) | 175 (170-180) | 178 (170-184) |
| Body weight (kg) | 84 (70-124) | 75 (70-108) | 76.5 (62-92) |
| **Ultrasound** |  |  |  |
| Right Testicular volume (ml) | 7.32 (3.85-9.17) | 15.46 (10.19-20.53) | 6.82 (1.75-10.81) |
| Right Epididymal head thickness (mm) | 8.0 (4.4-11.8) | 7.9 (8.24-18.59) | 7.25 (3.9-10.2) |
| Left Testicular volume (ml) | 6.87 (3.9-10) | 12.31 (8.24-18.59) | 6.25 (1.71-11.7) |
| Left epididymal head thickness (mm) | 8.2 (6-17.9) | 7.15 (5.7-8.4) | 9.4 (5.6-27.8) |
| **Semen** |  | | |
| Volume (ml) | 3.6 (2.5-8) | 2.9 (1.4-8.5) | 2.55 (1-5.5) |
| pH value | 7.5 (7-7.8) | 7.5 (7.4-8.5) | 7.3 (7.2-7.7) |
| Sperm concentration (mio/ml) | 0 (0-0.01) | 0 (0-0.01) | 0.01 (0-0.01) |
| Peroxidase- positive Leukocytes (mio/ml) | 0 (0-0.2) | 0 (0-10.5) | 0.1 (0-3) |
| Fructose (µmol/ejaculate) | 51.25 (37.25-245.6) | 72.96 (9.28-92.95) | 18.02 (5.4-90.8) |
| Glucosidase (mU/ejaculate) | 52.8 (19.14-103.18) | 19.2 (8.85-85) | 38.86 (19.62-63.25) |
| Elastase (ng/ml) | 15 (10-190) | 97 (10-260) | 122 (20-741) |
| Zinc (µmol/ejaculate) | 10.23 (3.6-28.38) | 10.64 (0.9-42.5) | 15.25 (3.6-31.6) |
| **Hormones** |  | | |
| Albumin (g/l) | 47 (42.2-50) | 48.1 (44-51) | 47.3 (43.7-50.4) |
| PSA (ng/ml) | 0.52 (0.13-0.92) | 0.57 (0.28-1.03) | 0.7 (0.3-1) |
| FSH (mU/ml) | 23.2 (10-39.9) | 3.7 (1.6-9.8) | 24.7 (15.3-48.0) |
| LH (mU/ml) | 8.6 (2.5-17.1) | 3.8 (2.2-6.6) | 9.15 (3.9-14.0) |
| Prolactin (uIU/ml) | 227 (111-292) | 176 (95-196) | 153.5 (111-381) |
| Estradiol (pmol/l) | 27 (16-31) | 27 (17-52) | 34.5 (14-43) |
| Testosterone (nmol/l) | 281 (203-447) | 440 (237-527) | 397 (217-618) |
| Free Testosterone (pmol/l) | 6.64 (3.28-8.27) | 8.37 (4.79-10.07) | 6.53 (3.53-11.31) |
| SHBG (nmol/l) | 24.4 (14.1-47.5) | 30.9 (21-61.5) | 38.1 (15.3-45.2) |

Data are presented as median and interquartile range (IQR)
