# Supplementary material for: Proteomic biomarkers in seminal plasma as predictors of reproductive potential in azoospermic men
Source: Front Endocrinol (Lausanne). 2024 Apr 9;15:1327800. doi: 10.3389/fendo.2024.1327800 (PMC11035875; doi:10.3389/fendo.2024.1327800)
Supplement: Supplementary file 3 [file Table_2.docx]

**Suppl. Tab. 2:**

Detailed testicular histology of azoospermia patients.

| Sample ID | Score TESE top right | Score TESE middle right | Score TESE bottom right | Score Micro-TESE right | Score TESE top left | Score TESE middle left | Score TESE bottom left | Score Micro-TESE left |
| --- | --- | --- | --- | --- | --- | --- | --- | --- |
| MA1 | 1 | 0.9 | 2 | 0 | 0 | 0 | 0 | 0 |
| MA2 | 0 | 1 | 5 | 0.6 | 0 | 0.1 | 5 | 2 |
| MA3 | 0 | 0.1 | 2 | 1 | 4 | 2 | 0 | 0.6 |
| MA4 | 0 | 0 | 0 | 3 | 0 | 0 | 0 | 0.2 |
| MA5 | 2 | 0.2 | 0.7 | 0.6 | 0.9 | 0.09 | 0.5 | 0.5 |
| MA6 | 4 | 0.4 | 0.4 | 0.7 | 0 | 0 | 0 | 0 |
| MA7 | 6 | 2 | 0.7 | 0.7 | 4 | 0.8 | 0.4 | 2 |
| MA8 | 2 | 0.3 | 0 | 0 | 0 | 0 | 0 | 0 |
| OA1 | 10 | 10 | 7 | 10 | 9 | 9 | 10 | 10 |
| OA2 | 10 | 10 | 10 | 10 | 10 | 10 | 10 | 10 |
| OA3 | 10 | 10 | 10 | 10 | 10 | 10 | 10 | 10 |
| OA4 | 10 | 10 | 10 | 10 | 10 | 10 | 10 | 10 |
| OA5 | 10 | 10 | 10 | 10 | 10 | 10 | 10 | 10 |
| OA6 | 10 | 10 | 10 | 10 | Single testis | Single testis | Single testis | Single testis |
| OA7 | 10 | 7 | 10 | 10 | 10 | 10 | 10 | 10 |
| SCO1 | 0 | 0 | 0 | 0 | 0 | 0 | 0 | 0 |
| SCO2 | 0 | 0 | 0 | 0 | 0 | 0 | 0 | 0 |
| SCO3 | 0 | 0 | 0 | 0 | 0 | 0 | 0 | 0 |
| SCO4 | 0 | 0 | 0 | 0 | 0 | 0 | 0 | 0 |
| SCO5 | 0 | 0 | 0 | 0 | 0 | 0 | 0 | 0 |
| SCO6 | 0 | 0 | 0 | 0 | 0 | 0 | 0 | 0 |
| SCO7 | 0 | 0 | 0 | 0 | 0 | 0 | 0 | 0 |

Scores were determined as described in Fietz and Kliesch, 2022 (Number of tubules containing elongated spermatids divided by the total number of tubules examined, multiplied by 10). Testis specimens were collected by experienced surgeons during microsurgical testicular sperm extraction (m-TESE) and immediately fixed in Bouin’s solution pending histological evaluation.
